# The diagnostic value of metagenomic next-generation sequencing for identifying *Streptococcus pneumoniae* in paediatric bacterial meningitis

**DOI:** 10.1186/s12879-019-4132-y

**Published:** 2019-06-04

**Authors:** Xi-xi Zhang, Ling-yun Guo, Lin-lin Liu, Ao Shen, Wen-ya Feng, Wen-hua Huang, Hui-li Hu, Bing Hu, Xin Guo, Tian-ming Chen, He-ying Chen, Yong-qiang Jiang, Gang Liu

**Affiliations:** 1 Key Laboratory of Major Diseases in Children, Ministry of Education, Department of Infectious Diseases, Beijing Children’s Hospital, Capital Medical University, National Center for Children’s Health, No. 56 Nan Lishi Road, Beijing, 100045 China; 2 Tianjin Medical Laboratory, BGI-Tianjin, Tianjin, China; 3 State Key Laboratory of Pathogen and Biosecurity, Institute of Microbiology and Epidemiology, Academy of Military Medical Science, Beijing, 100071 China

**Keywords:** Metagenomic next-generation sequencing (mNGS), Sensitivity, Specificity, *Streptococcus pneumonia*, Meningitis, Children

## Abstract

**Background:**

There is currently no research on the diagnostic value of metagenomic next-generation sequencing (mNGS) for a single pathogens in CSF. The aim of this study was to analyse the value of mNGS for identifying *Streptococcus pneumoniae* (*S. pneumoniae*) in paediatric bacterial meningitis.

**Methods:**

Bacterial meningitis (BM) cases from October 23, 2014, to December 31, 2016, and December 1, 2017, to July 31, 2018 at Beijing Children’s Hospital were reviewed. Clinical features and pathogens were analysed.

**Results:**

We diagnosed 135 patients with BM in this study. A total of 43 *S. pneumoniae* were identified by combination methods. 26/135 (19.3%) patients had positive results in *S. pneumoniae* by blood and/or cerebrospinal fluid (CSF) culture. Alere BinaxNow®*Streptococcus pneumoniae* Antigen test was positive in 35/135(25.9%) cases. 32/135 (23.7%) *S. pneumoniae* were identified by mNGS. Six CSF samples were identified as *S. pneumoniae* only by mNGS technology. Taking culture as the gold standard, the sensitivity and specificity of mNGS for diagnosing *S. pneumoniae* meningitis were 73.1 and 88.1%, respectively. The positive predictive value (PPV) and negative predictive value (NPV) of diagnosing *S. pneumoniae* meningitis by mNGS were 59.4 and 93.2%, respectively. When comparison between mNGS and combined tests (culture and Alere BinaxNow®*Streptococcus pneumoniae* Antigen test), the sensitivity and specificity of mNGS for *S. pneumoniae* identification were 70.3 and 93.9%, the PPV and NPV in the identification of *S. pneumoniae* by mNGS were 81.4 and 89.3%, respectively. The difference in number of unique reads of *S. pneumoniae*in from CSF sample (< 14 days onset) and CSF sample (> 14 days from onset) was statistically significant (170.5 VS. 13, *P* = 0.019). The difference in the collected time of CSF for culture and mNGS was statistically significant (4 days VS. 14 days, *P* < 0.001).

**Conclusions:**

mNGS has high sensitivity and specificity for *S. pneumoniae* identification. The pathogen load (number of unique reads) of *S. pneumonia* is related to the CSF collection time. mNGS was less affected than culture by the use of antibiotics before CSF collection.

## Highlights

Metagenomic Next-generation sequencing (mNGS) had high sensitivity and specificity for *S. pneumoniae* identification in CSF specimens.

mNGS was less affected than culture by the use of antibiotics before CSF collection.

The number of unique reads of *S. pneumoniae* detected by mNGS was related to the duration from onset to the sample collection time.

## Background

Bacterial Meningitis (BM) is an infection of the meninges and subarachnoid space that can sometimes present as an infection of the brain cortex and parenchyma [1]. BM had an annual incidence of 16 million people worldwide in 2013, of which 1.6 million had sequelaes [2]. BM causes significant morbidity and mortality in both developed and developing countries [3–5]. The fatality rate of *Streptococcus pneumoniae* (*S. pneumoniae*) meningitis can reach 20–37% in high-income countries and up to 51% in low-income countries [6]. Neurological sequelae are common among survivors [7–10]. Therefore, prompt pathogen diagnosis and accurate antibiotic treatment are essential to improve the prognosis of BM.

With the application of the meningococcal vaccine, *S. pneumoniae* conjugate vaccine and Hib vaccine, the incidence of BM caused by these pathogens has decreased in developed countries [11]. However, the pneumococcal conjugate vaccine is not universally used in China. A multicentre study of BM in Chinese children showed that *S. pneumoniae* was still the most common pathogen, occurring in up to 46.5% of cases [12]. The conventional pathogen detection method (culture) may take a long time (3 to 5 days) and have a low positive rate. Metagenomic next-generation sequencing (mNGS) is a high-throughput sequencing method that can directly detect the nucleic acids of pathogens in clinical specimens and then analyse the nucleic acid sequences by bioinformatics methods. As a novel diagnostic tool, mNGS has been used for the identification of various pathogens such as bacteria, viruses, fungi and parasites from clinical samples (tissues, CSF or plasma) in an unbiased, simultaneous and direct manner [13–20].

However, at present, there are few studies on pathogen detection by mNGS in bacterial meningitis. Our team previously identified pathogens from cerebrospinal fluid specimens in children with bacterial meningitis by mNGS. The results showed that mNGS is of great value for the identification of pathogens in cerebrospinal fluid (CSF), and *S. pneumoniae* is still the most common pathogen [17]. However, there is currently no research on the diagnostic value of mNGS for a single pathogen. Hence, this study aimed to investigate the diagnostic value of mNGS for identifying *S. pneumoniae* in children with BM.

## Methods

### Study population and specimen collection

All BM patients aged 29 days to 18 years were admitted to the Department of Infectious Diseases at Beijing Children’s Hospital from October 23, 2014, to December 31, 2016, and December 1, 2017, to July 31, 2018. The diagnosis criteria for BM were consistent with the World Health Organization (WHO), as follows: (1) acute fever (> 38.5 C rectal or > 38.0 C axillary); (2) headache, meningeal irritation, or altered consciousness; (3) at least one of the following in the cerebrospinal fluid: leukocytes > 100 cells/mm [3] or leukocytes 10–100 cells/mm [3] with elevated protein (> 100 mg/dL) or decreased glucose (< 40 mg/dL); and (4) positive culture, positive Gram stain, or positive bacterial antigen in the CSF. A case meeting diagnostic criteria 1, 2, and 3 at the same time was considered a probable case. A probable case meeting criterion 4 was considered a confirmed case. Exclusion criteria were as follows: cerebrospinal fluid < 1 ml and bloody CSF. The clinical data of all included cases including demographic characteristics, antibiotic use, specimen collection and clinical microbiology tests results [culture and Alere BinaxNow® *Streptococcus pneumoniae* Antigen test (Alere, USA)] were recorded.

One millilitre of CSF was collected for mNGS when the lumbar puncture was performed after admission. This study was approved by the Ethics Committee of Beijing Children’s Hospital affiliated to Capital Medical University (No. 2017–74). Written informed consent was obtained from the patient’s parents or other legal representatives.

### DNA extraction

DNA was extracted directly from the 300ul CSF sample (each patient and negative “no-template” control) using the TIANamp Micro DNA Kit (DP316, Tiangen Biotech, Beijing, China). The sample was added proteinase K (10 ml) and 300 ml buffer GB (with carrier RNA) and then incubated at 56 °C (10 min). After 300 ul cold absolute ethyl alcohol was added and the tube was incubated at room temperature (5 min). Transferring the liquid to a new adsorption column and the liquid was washed with buffer GD and buffer PW. The DNA was dissolved in 40ul of Tris–ethylenediaminetetraacetic acid buffer.

### Library generation, and sequencing

The extracted DNA was sonicated with a Bioruptor Pico device to generate 200–300 bp fragments. According to the standard protocol of the BGISEQ-500 sequencing platform (BGI-Tianjin, Tianjin, China), DNA libraries were constructed through end repaired, adapter added overnight and polymerase chain reaction amplification to the extracted DNA. Quality control was carried out using a bioanalyser (Agilent 2100, Agilent Technologies, Santa Clara, CA, USA) combined with quantitative PCR to measure the adapters before sequencing. DNA sequencing was then performed using the BGISEQ-500 platform (BGI-Tianjin, Tianjin, China) [21].

### Data processing and analysis

High quality sequencing data was generated after removal of short (< 35 bp) reads, low quality and low complexity reads. The readings were then mapped to the human reference genome (hg19 and YH sequences) using the Burrows-Wheeler Aligner [22]. The remaining data were aligned with the NCBI microbial genome database (ftp://ftp.ncbi.nlm.nih.gov/genomes/), which included the genome sequences of 3446 bacterial species (104 species of *Mycobacterium tuberculosis* and 45 species of mycoplasma/chlamydia), 1515 viral species, 206 fungal species and 140 parasites connected to human diseases. The mapped data were used for further analysis. The depth and coverage of each species was calculated using Soap Coverage on the SOAP website (http://soap.genomics.org.cn/).

### Quantitative real-time PCR (qPCR) validation

We performed *S. pneumonia* qPCR to validate the mNGS results. The DNA was extracted using the QIAamp cador Pathogen Mini Kit (Qiagen, 54,106). qPCR was carried out on the ViiATM 7 real-time PCR system using cycling conditions comprising 2 min at 50 °C and 10 min at 95 °C followed by 45 two-step cycles of 15 min at 95 °C and 1 min at 60 °C. The sequences of the primers and probes are as follows: F: ACGCAATCTAGCAGATGAAGCA, R: TCGTGCGTTTTAATTCCAGCT and P: FAM-AACGCTTGATACAGGGAG-MGB [23].

### Statistical methods

Continuous variables were expressed as the mean ± standard deviation or as the median. Two groups were compared using the independent t-test for parametric data and the Mann-Whitney *U* test for non-parametric data. Continuous variables with *P*-values < 0.05 were considered statistically significant, and all tests were 2-tailed. Based on the extracted data, a 2 × 2 contingency table was used to determine sensitivity, specificity, PPV, and NPV. All of the statistical analyses were conducted using SPSS 23.0 software (SPSS Inc. USA).

## Results

### Patient demographics and microbiology results

A total of 135 children with BM were included in this study. *S. pneumoniae* was identified in 43/135 (31.9%) patients by all microbiology tests. A total of 26/43 (60.5%) cases were male, and the median age was 11.5 (8.5, 48.9) months. A total of 37/135 (27.4%) cases were identified as *S. pneumonia* infection by clinical microbiology tests (culture and/or Alere BinaxNow® *Streptococcus pneumoniae* Antigen test). *S. pneumonia* was identified by blood and/or CSF culture in 26/135 (19.3%) cases. A total of 35/135 (25.9%) patients had positive results on the Alere BinaxNow® *Streptococcus pneumoniae* Antigen test. *S. pneumoniae* was identified in 32/135 (23.7%) patient CSF specimens by mNGS. Six CSF samples were identified as *S. pneumoniae* only by mNGS.

### mNGS information and the related influencing factors

**Fig. 1:**
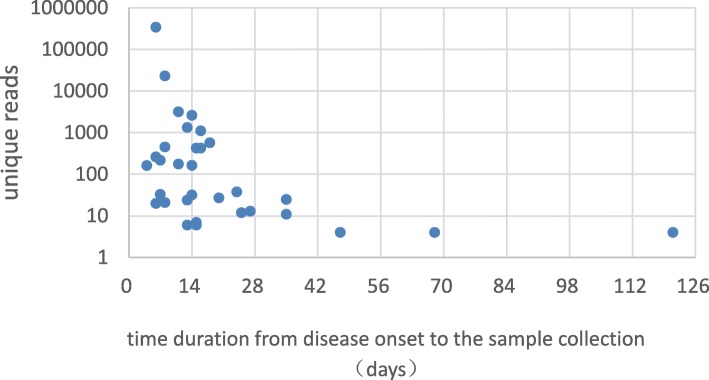
The relationship between unique reads of *Streptococcus pneumonia* and sample collection time

Among 32 patients who were diagnosed with *S. pneumoniae* meningitis by mNGS, the number of unique reads of *S. pneumonia* ranged from 4 to 341,303. The coverage of *S. pneumonia* ranged from 0.011 to 86.68%, with a depth value of 1–36.57 (Table 1). The collection time of CSF specimens for mNGS was 4 to 121 days after disease onset, and the median collection time was 14 days. The difference in the number of unique reads of *S. pneumoniae* in the different groups (CSF collection time < 14 days and > 14 days from onset) was statistically significant (170.5 vs. 13, *P* = 0.019) (Fig. 1). All 32 mNGS-positive children were treated with antibiotics prior to mNGS sample collection.

**Table 1:** Clinical microbiology results and sequencing information for patients who were positive for *S. pneumonia* by mNGS

	Clinical microbiology tests			mNGS				qPCR
Patient	Pathogen	Culture (blood/CSF)	Antigen test	Pathogen	Reads	Coverage%	Depth	
P1	SP	–	+	SP	262	1.1	1	ND
P2	SP	+	+	SP	1105	11	1.1	ND
P3	SP	+	+	SP	573	2.6	1	+
P4	SP	+	+	SP	429	1.9	1	+
P5	SP	–	+	SP	425	1.9	1	+
P6	SP	–	+	SP	176	1.8	1	ND
P7	SP	+	ND	SP	162	0.75	1	+
P8	SP	+	+	SP	38	0.16	1	ND
P9	SP	–	+	SP	21	0.12	1	ND
P10	SP	+	+	SP	13	0.066	1	ND
P11	SP	+	+	SP	11	0.052	1	ND
P12	SP	+	+	SP	7	0.048	1	–
P13	SP	+	+	SP	6	0.034	1	–
P14	SP	+	+	SP	4	0.041	1	ND
P15	SP	–	+	SP	4	0.016	1	–
P16	SP	–	+	SP	3189	28	1.3	ND
P17	SP	–	+	SP	23,247	75	3.9	ND
P18	SP	+	+	SP	25	0.19	1	+
P19	SP	+	+	SP	2603	7.25	1.06	ND
P20	SP	+	+	SP	341,303	86.68	36.57	+
P21	SP	+	+	SP	165	0.7413	1.01	+
P22	SP	+	+	SP	1335	12.03	1.09	+
P23	SP	+	+	SP	456	5.41	1.04	+
P24	SP	+	+	SP	32	0.0744	1	ND
P25	SP	+	+	SP	218	1.04	1	ND
P26	SP	+	+	SP	20	0.1488	1	–
P27	–	–	–	SP	33	0.31	1	ND
P28	SA	SA	ND	SP	27	0.15	1	–
P29	–	–	–	SP	24	0.12	1	ND
P30	–	–	ND	SP	12	0.95	1	ND
P31	–	–	–	SP	6	0.055	1	ND
P32	–	–	–	SP	4	0.011	1	–

Note: *mNGS* metagenomic next-generation sequencing, *CSF* Cerebrospinal fluid, *qPCR* quantitative real-time PCR, *SP Streptococcus pneumonia, SA Streptococcus aureus, E. coli Escherichia coli, +* positive, *−* negative, ND Not done

Based on the number of unique reads of *S. pneumoniae*, 32 patients were divided into two groups. The unique reads ≤100 group comprised 17 (41.4%) patients, while the unique reads > 100 group comprised 15 patients. Comparing the two groups, patients in the unique reads > 100 group had a generally shorter duration from disease onset to sample collection than patients in the mNGS unique reads ≤100 group (11 days vs. 20 days, *P* = 0.027). There was a significant difference in the simultaneous CSF white blood cell count and protein level between the two groups (10 × 10^9^/L vs. 32 × 10^9^/L, *P* = 0.001; 1280 mg/L vs. 855 mg/L, *P* = 0.016) (Table 2).

**Table 2:** Comparison of different mNGS unique read groups

Items	mNGS unique reads ≤ 100 (*N* = 17)	mNGS unique reads > 100 (*N* = 15)	*P*
Sex (female/male)	12/5	7/8	0.169
Age (months)	11.2(5.9,33.5)	13.9(9.2,48.9)	0.290
Duration from onset to CSF collection (days)	20(13,35)	11(7,16)	***0.027***
Fever [n (%)]	17(100)	15(100)	
Seizure [n (%)]	8(47.1)	10(66.7)	0.265
Vomiting [n (%)]	10(58.8)	9(60)	0.946
Meningeal irritation [n (%)]	9(52.9)	12(80)	0.103
Altered mental status [n (%)]	7(41.2)	8(53.3)	0.492
Peripheral blood			
WBC (×10^9^/L)	16.98(15.10,23.69)	17.3(11.9,24,2)	0.895
Neutrophils (%)	78.2(60.7,86.3)	77.8(62.7,85.6)	0.985
Haemoglobin (g/L)	104.4 ± 19.0	105.5 ± 18.7	0.767
C-reactive protein (mg/L)	63.5(19.6104)	109.5(49,157.4)	0.281
1st CSF^a^			
WBC (×10^6^/L)	1714(495,2635)	1886(910,3600)	0.597
Protein (mg/L)	1280(779,2250)	1650(1161,3033)	0.180
Glucose (mmol/L)	1.84(1.00,3.34)	0.67(0.19,1.92)	0.081
Simultaneous CSF^b^			
WBC (×10^6^/L)	10 (2,12)	32(14,76)	***0.001***
Protein (mg/L)	1280(779,2250)	855(527,1488)	***0.016***
Glucose (mmol/L)	3.42 ± 0.99	3.39 ± 1.12	0.928

Note: ^a^The first CSF refers to the first cerebrospinal fluid examination in the early stage of the disease

^b^Simultaneous CSF refers to the CSF results by clinical testing at which time CSF was also tested by mNGS

*P*-values in bold italic shows variables with evidence of association in univariate analysis

### Comparison of mNGS and clinical microbiology methods

The median collection time of CSF specimens for culture that had positive *S. pneumoniae* results was 4 (3, 5) days. The median collection time of CSF specimens for mNGS that had positive *S. pneumoniae* results was 14 (8, 23) days from onset. The difference in the collected time of CSF for culture and mNGS was statistically significant (4 days vs. 14 days, *P* < 0.001).

Among the 135 BM patients, mNGS and clinical microbiology tests (culture and/or Alere BinaxNow® *Streptococcus pneumoniae* Antigen test) were both positive for *S. pneumonia* in 26 (19.3%) samples and were both negative for *S. pneumonia* in 92 (68.1%) samples. The accordance rate of mNGS and clinical microbiology tests (culture and/or Alere BinaxNow® *Streptococcus pneumoniae* Antigen test) for the identification of *S. pneumoniae* was 87.4% (118/135).

The comparison between mNGS and clinical microbiology tests (culture and Alere BinaxNow® *Streptococcus pneumoniae* Antigen test) was shown in Table 3. Taking culture as the gold standard, the sensitivity and specificity of *S. pneumoniae* identification by mNGS were73.1 and 88.1%, respectively. The PPV and NPV of diagnosing *S. pneumoniae* meningitis by mNGS were59.4 and 93.2%, respectively. Comparing mNGS and the combined tests (culture and Alere BinaxNow® *Streptococcus pneumoniae* Antigen test), the sensitivity and specificity of mNGS for *S. pneumoniae* identification were70.3 and 93.9%, respectively, and the PPV and NPV for the identification of *S. pneumoniae* by mNGS were 81.4 and 89.3%, respectively.

**Table 3:** Diagnostic performance of mNGS compared with culture and Alere BinaxNow® *Streptococcus pneumoniae* Antigen test for the detection of *Streptococcus pneumoniae*

Clinical microbiology tests	mNGS			
	Sensitivity (%)	Specificity (%)	PPV (%)	NPV (%)
Culture^a^	73.1	88.1	59.4	93.2
Combined test^b^	70.3	93.9	81.3	89.3

Note: ^a^refers to the blood and/or CSF culture; ^b^refers to culture and or Alere BinaxNow® *Streptococcus pneumoniae* Antigen test. *PPV* positive predictive value, *NPV* negative predictive value

In total, we performed qPCR validation for 15 CSF specimens, while insufficient CSF sample was available for qPCR validation in the other cases. These 15 CSF specimens were collected for mNGS and qPCR at the same time. The qPCR results were positive in 9 (9/15, 60%) cases [(mNGS unique reads: 429 (25–341,303)] and negative in 6 (6/15, 40%) cases. The latter 6 cases had fewer unique reads of *S. pneumonia,* and the number of unique reads was 4, 4, 6, 7, 20 and 27. The clinical microbiology tests (culture and/or Alere BinaxNow® *Streptococcus pneumoniae* Antigen test) were positive for *S. pneumonia* in 4 (4/6) cases (unique reads: 4, 6, 7 and 20). One (1/6) case (unique reads: 4) was negative for *S. pneumonia* in the clinical microbiology tests (blood and/or CSF culture and Alere BinaxNow® *Streptococcus pneumoniae* Antigen test). Another case (1/6) (P28, unique reads: 27) was positive for *Staphylococcus aureus* (*S. aureus*) in the blood culture. However, the result of specific PCR from CSF specimens was negative for both *S. pneumoniae* and *S. aureus* in P28.

In addition, we identified multiple pathogens in the CSF sample of P14 by mNGS, including *Escherichia coli* (*E. coli*) (unique reads 30) and *S. pneumoniae* (unique reads 4). In terms of clinical microbiology, P14 had a positive result for *S. pneumoniae* in the CSF culture and a positive Alere BinaxNow® *Streptococcus pneumoniae* Antigen test in the CSF. However, the Sanger validation of the CSF sample was negative for both *S. pneumoniae* and *E. coli.*

## Discussion

In this study, clinical microbiology tests (culture and Alere BinaxNow®*Streptococcus pneumoniae* Antigen test) were compared with an emerging pathogen detection method (mNGS). When the combined culture and pneumococcal antigen test were regarded as the gold standard, mNGS showed high sensitivity and specificity for the diagnosis of *S. pneumonia* meningitis. Previous studies have reported that mNGS has different sensitivities and specificities for the identification of different types of pathogens (bacterial, viral or fungal). The sensitivity and specificity of mNGS for the identification of bacteria had a wide range, with sensitivity ranging from 50.7 to 100% and specificity ranging from 76.5 to 87.5% [16, 24]. In this study, some of the CSF specimens were collected at the convalescence stage. If all CSF specimens were collected during the acute phase, the sensitivity of mNGS would be higher.

The number of unique reads of *S. pneumoniae* from CSF collected ≤14 days from the disease onset was significantly higher than those from CSF collected > 14 days from the disease onset. Patients in the unique reads > 100 group had a generally shorter duration from disease onset to the sample collection than those in the unique reads ≤100 group. These results together suggest that the number of unique reads of *S. pneumoniae* detected by mNGS was associated with specimen collection time, which is consistent with a previous study showing that with the use of effective antibiotics and disease improvement, the number of unique reads of pathogens decreased [25].

In this study, we observed that the time from disease onset to CSF collection was longer for mNGS than for culture. All patients were given antibiotics prior to mNGS and culture, and all patients were treated with antibiotics after the onset of disease. This phenomenon suggested that after antibiotic use, the timespan for positive pathogen identification by mNGS was longer than the timespan for positive pathogen identification by culture. A previous study suggested that the DNA of the pathogen may survive in body fluids for a prolonged period of time, and mNGS was less affected than culture by the use of antibiotics before CSF collection [24, 26, 27].

Among the 6 CSF samples that were negative for *S. pneumonia* by qPCR, *S. pneumonia* was identified in 4 (4/6) samples by both mNGS and clinical microbiology tests (blood and/or CSF culture and Alere BinaxNow®*Streptococcus pneumoniae* Antigen test)*.* This result showed that mNGS appears to have a higher sensitivity than qPCR in the test cases. *S. pneumonia* (unique reads 27) was identified in P28 CSF sample by mNGS, while *S. aureus* was detected by blood culture. The P28 CSF sample was found to be negative for both *S. pneumoniae* and *S. aureus* by qPCR. In many mNGS systems, short read sequences lead to difficulties in assembling and matching to the reference sequence, especially in repeat regions, resulting in difficulties defining the final pathogen. Therefore, when the number of unique reads is very small, the interpretation of pathogen data needs to be very careful and cautious [28].

We compared groups with ≤100 unique reads and groups with > 100 unique reads and analysed factors that could affect the number of unique reads of *S. pneumoniae*. There were significant differences in the simultaneous CSF white blood cell counts and protein levels between the two groups. These results suggested that the inflammation status during simultaneous mNGS detection might determine the number of unique reads of the pathogen. The number of unique reads may indirectly monitor disease progression.

Currently, the criteria for diagnosing single pathogens by mNGS are unclear. Different studies have reported different diagnostic criteria, mainly based on the coverage rate, the relative abundance of pathogens or unique reads of pathogens [16, 29].

In this study, the minimum number of unique reads of *S. pneumoniae* was 4, and the clinical microbiology test was also positive for *S. pneumoniae*. Therefore, the positive criteria for single pathogen identification by mNGS testing require further research by a larger sample size.

There were several limitations in this study. First, the collection time of some CSF samples for mNGS and clinical microbiology tests was different. The collection time of CSF was later for mNGS than for the clinical microbiology tests, which may affect the positive identification rate of mNGS. Some of the CSF specimens were collected at the convalescence stage. Second, this study was retrospective and had a limited number of BM cases, and thus large-scale research is needed. Third, mNGS still has low sequencing depth, and the pathogen database is imperfect [30]. However, sequencing technology is developing rapidly, and it is believed that the depth of sequencing will improve within a short period of time. mNGS can further improve the sensitivity and specificity of pathogen identification [31].

## Conclusion

This study is the first to analyse the diagnostic value of mNGS for the identification of a single pathogen (*S. pneumoniae*). mNGS has high sensitivity and specificity for *S. pneumoniae* identification. Our study also demonstrated the relationship between the pathogen load (unique reads) and CSF collection time. Although there are no uniform criteria for pathogen identification by mNGS, there are many difficulties in interpreting the mNGS results. With the development of mNGS technology, mNGS could be a promising alternative diagnostic tool for pathogen detection.

## Data Availability

The datasets analysed during the current study are available from the corresponding author on reasonable request.

## Abbreviations

BM: bacterial meningitis
CSF: cerebrospinal fluid
mNGS: metagenomic next-generation sequencing
NPV: negative predictive value
PPV: positive predictive value
qPCR: Quantitative real-time PCR
*S. pneumoniae*: *Streptococcus pneumonia*
*SA*: *Staphylococcus aureus*
